# Visual hallucinations and inferior longitudinal fasciculus in Parkinson's disease

**DOI:** 10.1002/brb3.1883

**Published:** 2020-10-20

**Authors:** Natsuko Yuki, Akira Yoshioka, Ryo Mizuhara, Tadashi Kimura

**Affiliations:** ^1^ Department of Neurology National Hospital Organization Maizuru Medical Center Maizuru Japan; ^2^ Department of Neurology Kyoto Kizugawa Hospital Joyo Japan; ^3^ Department of Clinical Research National Hospital Organization Maizuru Medical Center Maizuru Japan

**Keywords:** diffusion tensor imaging, inferior longitudinal fasciculus, Parkinson's disease, visual hallucination, white matter

## Abstract

**Introduction:**

We investigated whether disruption of the inferior longitudinal fasciculus and inferior fronto‐occipital fasciculus are associated with visual hallucinations in Parkinson's disease (PD).

**Methods:**

Sixty consecutive right‐handed patients with PD with and without visual hallucinations were enrolled in this cross‐sectional study. Diffusion tensor imaging was acquired by 3.0 T magnetic resonance imaging. We measured fractional anisotropy and mean diffusivity of the bilateral inferior longitudinal fasciculus and inferior fronto‐occipital fasciculus using diffusion tensor tractography analysis software.

**Results:**

Seventeen patients with PD had visual hallucinations; these patients tended to have lower fractional anisotropy and higher mean diffusivity values in all fasciculi than did patients without visual hallucinations. A univariate logistic analysis showed that the presence of visual hallucinations was significantly associated with lower fractional anisotropy and higher mean diffusivity of the left inferior longitudinal fasciculus, and lower Mini‐Mental State Examination (MMSE) scores. A multivariable logistic analysis adjusted by MMSE scores and disease duration showed a significant association between the presence of visual hallucinations and fractional anisotropy and mean diffusivity values of the left inferior longitudinal fasciculus.

**Conclusions:**

Our results suggest that disruption of left inferior longitudinal fasciculus integrity is associated with visual hallucinations in patients with PD, independent of cognitive impairment and disease duration.

## Graphical abstract

We investigated whether disruption of the inferior longitudinal fasciculus is associated with visual hallucinations in Parkinson's disease (PD) used by diffusion tensor imaging. A multivariable logistic analysis adjusted by MMSE scores showed a significant association between the presence of visual hallucinations and fractional anisotropy and mean diffusivity values of the left inferior longitudinal fasciculus. Our results suggest that disruption of left inferior longitudinal fasciculus integrity is associated with visual hallucinations in patients with PD, independent of cognitive impairment.

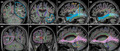

## 1 INTRODUCTION

Visual hallucinations (VH) are one of the most common nonmotor symptoms in patients with Parkinson's disease (PD). While the pathophysiology of VH in PD has not been clarified, changes in visual perceptual function seem to be associated with VH in PD. Recent studies have reported that VH in PD was associated with atrophy in the lateral geniculate body (Lee et al., 2016), dysfunction in the dorsal and ventral visual pathways (Auning et al., 2014; Boecker et al., 2007; Gallagher et al., 2011; Goldman et al., 2014; Guimarães et al., 2018; Harding et al., 2002; Onofrj et al., 2013), frontal lobe dysfunction (Onofrj et al., 2013; Ramírez‐Ruiz et al., 2008), impairment in bottom‐up visual perceptual processing (Connor et al., 2004; Ibarretxe‐Bilbao et al., 2011; Meppelink et al., 2009; Stebbins et al., 2004), and connectivity disruptions in the ventral and dorsal attention networks and the Default Mode Network (Shine et al., 2011, 2015; Shine et al., 2014; Yao et al., 2014).

The inferior longitudinal fasciculus (ILF) is a major associative tract that connects the occipital and anterior temporal lobes and is topographically well located to subserve the ventral visual pathway (Catani et al., 2003; Herbet et al., 2018). The ILF has multiple functions, including visual‐related processes such as objective and facial recognition, reading, regulation of lexical and semantic processes, and emotional regulation (Haghshomar et al., 2018; Herbet et al., 2018). One diffusion tensor imaging (DTI) study reported that the disruption of white matter integrity in the ILF was associated with VH in patients with dementia with Lewy bodies (DLB) (Kantarci et al., 2010). In patients with PD, it has been reported that the disruption of the integrity of the ILF was associated with cognitive impairment, color discrimination deficits, mood disorders, and tremor‐dominant motor symptoms (Haghshomar et al., 2018). However, the involvement of the ILF in patients with PD with VH has not yet been investigated.

The ILF has anatomical contacts with other white matter pathways, such as the inferior fronto‐occipital fasciculus (IFOF) (Herbet et al., 2018). The IFOF forms the sagittal stratum where the ILF passes through the temporal lobe, and both the ILF and IFOF terminate in the posterior occipitotemporal lobe (Herbet et al., 2018). It has been suggested that the IFOF might play an important role in visual processing, such as facial recognition and spatial cognition, much like the ILF (Herbet et al., 2017, 2018; Wu et al., 2016). Therefore, we investigated the involvement of both the ILF and IFOF in PD patients with and without VH using DTI.

## 2 MATERIALS AND METHODS

### 2.1 Patients

This was a cross‐sectional study. We enrolled 60 consecutive right‐handed patients with PD who underwent DTI from April 2017 to March 2019.

A clinical diagnosis of PD was made according to the United Kingdom PD Society Brain Bank diagnostic criteria (Hughes et al., 1992). Exclusion criteria were as follows: (a) the presence of other parkinsonian syndromes (Parkinson‐plus syndromes, and vascular and drug‐induced parkinsonism); (b) the presence of DLB; (c) contraindications to magnetic resonance imaging (MRI); and (d) normal ^123^I‐ioflupane uptake.

Visual hallucinations in this study were defined as complex VH, which means that persons, animals, and objects were perceived in the absence of an external stimulus. Patients with minor hallucinations and drug‐induced VH were excluded. We defined minor hallucinations as sense of presence, passage hallucinations and visual illusions. We defined drug‐induced VH as VH that occurred within 12 weeks after starting or increasing antiparkinsonian drugs or VH that occurred within 12 weeks after withdrawal or reduction of antiparkinsonian drugs. Patients with a history of recurrent complex VH at any time between the onset of motor impairment and DTI examination were defined as having PD with VH (PD‐VH). In contrast, patients without a history of recurrent complex VH were defined as having PD without VH (PD‐non‐VH).

The severity of motor dysfunction was evaluated using the Hoehn and Yahr (H&Y) stage. Cognitive function was evaluated using the Mini‐Mental State Examination (MMSE). Moreover, the duration of PD and levodopa equivalent daily dose (LLED) were evaluated.

This study was approved by the clinical study guidelines for the ethics committee at the National Hospital Organization Maizuru Medical Center. Written informed consent was obtained from patients and their families.

### 2.2 DTI acquisition

All patients underwent brain MRI examination on a 3.0 T system (Ingenia, Philips) using a 16‐channel head‐neck coil. DTI acquisition included a single‐shot multislice spin‐echo planar imaging sequence and the following parameters were used: repetition time (TR): 3,091 ms; echo time (TE): 91 ms; flip angle: 90°; field of view: 224 × 224 mm; and reconstruction voxel size: 2.33 × 2.38 × 2.50 mm. The acquisition consisted of 48 slices, and the scan time was 9 min 47 s.

### 2.3 Diffusion tensor tractography analysis

The collected DTI data were sent to a Philips Extended MR Workstation and analyzed using FiberTrak software (Philips). The step width of fiber tracking was set at 10 mm, and the condition of fiber tracking was a fractional anisotropy (FA) value of <0.15 and flip angle of >27°.

**FIGURE 1:**
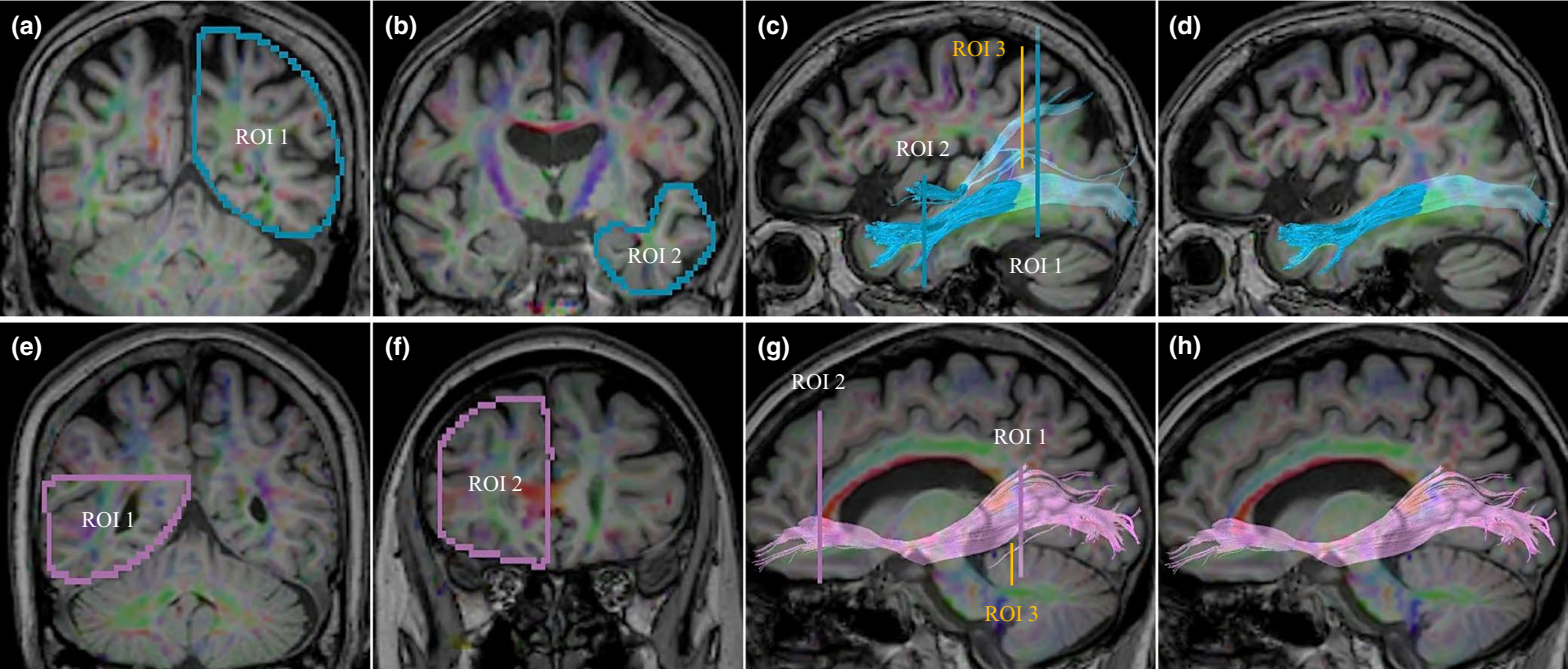
Identification of the inferior longitudinal fasciculus and inferior fronto‐occipital fasciculus. The reconstruction process for the left ILF (a–d) and the right IFOF (e–h). (a) The first ROI (ROI1) included the posterior termination of the left ILF. (b) The second ROI (ROI2) included the anterior termination of the left ILF. (c) The fiber bundle passing through ROI1 and ROI2. This fiber bundle involved not only the part of the ILF that connects the temporal and occipital lobes, but also other mixed fibers that connect the temporal and parietal lobes. To identify these other mixed fibers, the ROI to be excluded (ROI3) was drawn. (d) The pure left ILF without any mixed fibers. (e–h) The IFOF was identified in the same way as was the ILF. IFOF, inferior fronto‐occipital fasciculus; ILF, inferior longitudinal fasciculus; ROI, region of interest

The ILF and IFOF were identified using a region of interest (ROI) approach (Catani et al., 2002). Two ROIs for each region were selected using a standard textbook (Oishi et al., 2011) as a reference and were manually set over the region that included the posterior and anterior termination of the ILF and IFOF. To reconstruct the ILF, the first ROI was drawn on the entire hemisphere on a coronal slice at the posterior edge of the cingulum, and the second ROI was drawn on the entire temporal lobe in the most posterior slice in which the temporal lobe was connected to the frontal lobe. To reconstruct the IFOF, the first ROI was drawn on the occipital lobe on a coronal slice at the midlevel of parieto‐occipital sulcus, and the second ROI was drawn on the anterior genu of the corpus callosum covering the frontal lobe trajectories. Then, to exclude fiber bundles that contained any fibers that were not involved in the ILF or IFOF, the ROI to erase the fiber bundle was manually set in the regions involving other mixed fibers. As an example, the reconstruction process for the left ILF is shown in Figure 1.

We evaluated the FA values and the mean diffusivity (MD) values of the voxels containing each fiber bundle on the right and left sides. All analyses were performed by two neurologists, and data were anonymous.

### 2.4 Statistical analysis

We used Mann–Whitney U tests to compare the means of continuous variables (age, MMSE scores, H&Y stage, disease duration, and LLED) and chi‐square tests to compare the proportions of categorical variables (sex) between the PD‐VH and PD‐non‐VH groups. We used univariate and multivariable logistic regression analyses to evaluate the association between the presence of VH and DTI measurements. MMSE score and disease duration were included as confounding factors in the multivariable logistic regression analyses because VH in PD is associated with cognitive impairment and disease duration (Lee et al., 2012; Wada‐Isoe et al., 2008). First, univariate logistic regression analyses were performed under the following conditions: (a) The dependent variable was the presence of VH, and (b) the independent variables were clinical characteristics (age, sex, MMSE scores, H&Y stage, disease duration, LLED, and DTI measurements (FA and MD values of the bilateral ILF and IFOF)). Next, multivariable logistic regression analyses adjusted by MMSE scores and disease duration were performed under the following conditions: (a) The dependent variable was the presence of VH, and (b) the independent variables were FA and MD values. The threshold for significance was *p* < .05. All statistical analyses were conducted using SPSS (version 25.0; IBM Corporation).

## 3 RESULTS

Of the 60 patients with PD, 17 had VH. Clinical characteristics of the PD‐non‐VH and PD‐VH groups are listed in Table 1. There were no significant differences in age, sex, H&Y stage, or LLED between the PD‐non‐VH and PD‐VH groups. The median MMSE scores of the PD‐non‐VH and PD‐VH groups were 27.0 (interquartile range [IR] 24.8–30.0) and 24.0 (IR 21.0–27.0), respectively, and the MMSE score was significantly lower in the PD‐VH group than in the PD‐non‐VH (*Z*‐value = −2.684, *p* = .008). The median disease duration of the PD‐non‐VH and PD‐VH groups were 6.1 (IR 3.0–10.1) and 10.5 (IR 8.0–12.7) years, respectively, and this was significantly longer in the PD‐VH group than in the PD‐non‐VH group (*Z*‐value = −2.486, *p* = .013). The median LLEDs in the PD‐non‐VH and PD‐VH groups were 300 (IR 200–400) and 400 (IR 288–475) mg, respectively. Although this difference was not significant, the PD‐VH group tended to have a larger dose of L‐dopa than the PD‐non‐VH group (*Z*‐value = −1.782, *p* = .08).

**TABLE 1:** Clinical characteristics of patients with Parkinson's disease with and without visual hallucinations

Variable	PD‐non‐VH (*n* = 43)	PD‐VH (*n* = 17)	*Z*‐value	*χ^2^*	*p*‐value
Age, years	78.0 (70.0–82.0)	75.0 (70.0–77.0)	−0.838		.40^a^
Female, *n* (%)	26 (60)	11 (65)		0.093	.76^b^
MMSE score	27.0 (24.8–30.0)	24.0 (21.0–27.0)	−2.684		.008^a^
H&Y stage	3.0 (3.0–3.0)	3.0 (3.0–3.0)	−0.487		.63^a^
Disease duration, years	6.1 (3.0–10.1)	10.5 (8.0–12.7)	−2.486		.013^a^
LEDD, mg	300 (200–400)	400 (288–475)	−1.782		.08^a^

Note

Values shown are the median (interquartile range) unless otherwise specified.

Abbreviations: H&Y, Hoehn & Yahr; LLED, levodopa equivalent daily dose; MMSE, Mini‐Mental State Examination; PD‐non‐VH, patients with Parkinson's disease without visual hallucinations; PD‐VH, patients with Parkinson's disease with visual hallucinations.

^a^ Mann–Whitney *U* test.

^b^ Fisher's exact test.

**FIGURE 2:**
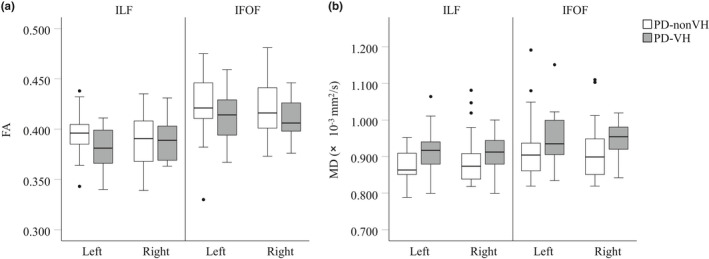
Fractional anisotropy and mean diffusivity values in patients with Parkinson's disease with and without visual hallucinations. (a) FA values. There was a tendency toward lower FA values in the PD‐VH group than in the PD‐non‐VH group in the left ILF and bilateral IFOF. (b) MD values. There was a tendency toward higher MD values in the PD‐VH group than in the PD‐non‐VH group in the bilateral ILF and IFOF. FA, fractional anisotropy; IFOF, inferior fronto‐occipital fasciculus; ILF, inferior longitudinal fasciculus; MD, mean diffusivity; PD, Parkinson's disease; VH, visual hallucinations

The DTI results are shown in Figure 2. There was a tendency toward lower FA values in the PD‐VH group than in the PD‐non‐VH group in the left ILF and bilateral IFOF (Figure 2a). There was a tendency toward higher MD values in the PD‐VH group than in the PD‐non‐VH group in the bilateral ILF and IFOF (Figure 2b). Univariate and multivariable logistic regression analyses were performed to evaluate the correlation between the FA and MD values and the presence of VH (Table 2). The univariate logistic regression analysis showed that the presence of VH was significantly associated with a lower MMSE score (odd ratio [OR] = 0.985; 95% confidence intervals [CI] = 0.640–0.939; *p* = .009), lower FA values in the left ILF (OR = 0.000; 95% CI = 0.000–0.009; *p* = .02), and higher MD values in the left ILF (OR = 8.157 × 107; 95% CI = 59.075–1.126 × 1,014; *p* = .012). A multivariable logistic regression analysis adjusted by MMSE scores and disease duration showed that the presence of VH was significantly associated with lower FA values (OR = 0.000; 95% CI = 0.000–0.208; *p* = .04) and higher MD values (OR = 5.369 × 10^6^; 95% CI = 1.142–2.525 × 10^13^; *p* = .048) in the left ILF.

**TABLE 2:** Correlations between clinical characteristics, fractional anisotropy and mean diffusivity values, and visual hallucinations evaluated using univariate and multivariable logistic regression analysis adjusted by Mini‐Mental State Examination scores and disease duration

	Univariate analysis			Multivariable analysis adjusted for MMSE score and disease duration		
	*B*	OR (95% CI)	*p*‐value	*B*	OR (95% CI)	*p*‐value
Age, years	−0.015	0.985 (0.914–1.061)	.68	–	–	–
Male	−0.181	0.834 (0.260–2.681)	.76	–	–	–
H&Y	0.426	1.531 (0.548–4.275)	.42	–	–	–
MMSE	−0.255	0.775 (0.640–0.939)	.009*	–	–	–
Disease duration, years	0.077	1.080 (0.980–1.191)	.12	–	–	–
LLED	0.003	1.003 (1.000–1.007)	.09	–	–	–
FA						
Left ILF	−36.386	0.000 (0.000–0.009)	.02*	−36.004	0.000 (0.000–0.208)	.04*
Right ILF	4.618	101.251 (0.000–6.714 × 10^12^)	.72	16.877	2.136 × 10^7^ (0.000–1.599 × 10^21^)	.30
Left IFOF	−16.539	0.000 (0.000–78.662)	.12	−15.264	0.000 (0.000–2.558 × 10^3^)	.20
Right IFOF	−15.013	0.000 (0.000–5419.715)	.21	−13.361	0.000 (0.000–2.811 × 10^6^)	.30
MD						
Left ILF	18.217	8.157 × 10^7^ (59.075–1.126 × 10^14^)	.012*	15.496	5.369 × 10^6^ (1.142–2.525 × 10^13^)	.048*
Right ILF	8.304	4,040.514 (0.220–7.425 × 10^7^)	.10	5.828	339.641 (0.002–5.608 × 10^7^)	.34
Left IFOF	7.276	1,444.553 (0.550–3.800 × 10^6^)	.07	6.892	984.715 × 10^2^ (0.150–6.465 × 10^6^)	.12
Right IFOF	8.520	5,015.933 (0.588–4.278 × 10^7^)	.07	4.166	64.429 (0.002–1.670 × 10^6^)	.42

Abbreviations: CI, confidence interval; DTI, diffusion tensor imaging; FA, fractional anisotropy; IFOF, inferior fronto‐occipital fasciculus; ILF, inferior longitudinal fasciculus; LLED, levodopa equivalent daily dose; MD, mean diffusivity; MMSE, Mini‐Mental State Examination; OR, odds ratio.

* *p* < .05.

## 4 DISCUSSION

We investigated the involvement of ILF and IFOF in PD patients with VH using DTI. Multivariable logistic regression analyses adjusted by MMSE scores and disease duration showed that the presence of VH was significantly associated with lower FA and higher MD values in the left ILF. These results suggest that a disruption of ILF integrity is associated with VH in PD, independent of cognitive impairment and disease duration.

We found no significant between‐group differences in age, sex, H&Y stage, or LLED. On the other hand, the PD‐VH group had significantly lower MMSE scores and a significantly longer disease duration than the PD‐non‐VH group. Although not significant, the PD‐VH group tended to have larger doses of L‐dopa than the PD‐non‐VH group. Previous studies have shown that VH in PD was associated with cognitive impairment, a longer disease duration, and higher L‐dopa levels (Fénelon et al., 2000; Lee et al., 2012; Wada‐Isoe et al., 2008; Zhu et al., 2017). In this study, univariate logistic regression analyses showed that the presence of VH was only significantly associated with MMSE scores and no other clinical characteristics.

We found that disruption of left ILF integrity, but not of IFOF integrity, was associated with the presence of VH in patients with PD. The ILF connects the occipital lobes with the anterior temporal lobes. Recent MRI studies have reported atrophy of the temporo‐occipital regions in PD‐VH (Goldman et al., 2014; Lee et al., 2017). These regions are involved in visual recognition based on stimulus saliency (a bottom‐up process) and the ventral visual stream, which underlies object recognition. Structural and functional MRI studies have reported that bottom‐up processes are impaired in PD‐VH (Ibarretxe‐Bilbao et al., 2011; Meppelink et al., 2009), and several neuroimaging and pathological studies have indicated that changes in the ventral visual stream are associated with VH in PD (Boecker et al., 2007; Firbank et al., 2018; Gallagher et al., 2011; Goldman et al., 2014). Impairment of the bottom‐up process and ventral visual stream could be a result of the neuropathological increase in Lewy body deposits in the temporal lobes in PD‐VH (Gallagher et al., 2011; Harding et al., 2002; Papapetropoulos et al., 2006). A previous DTI study reported that a disruption in the ILF accompanied by amygdala diffusivity changes was associated with VH in patients with DLB (Kantarci et al., 2010). The authors suggested that a disruption in the ILF might be related to a retrograde degeneration of the axonal projections that connect the amygdala to the visual association cortex. Therefore, our results indicate that ILF disruption is associated with the genesis of VH in PD.

While there was a tendency toward lower FA values and higher MD values in the bilateral IFOF of the PD‐VH group compared with the PD‐non‐VH group, the univariate logistic regression analysis revealed that the presence of VH was not significantly associated with a lower FA or a higher MD in the bilateral IFOF. Using pixel‐based analysis, Zarkali et al. (2020) recently reported that patients with PD and low visual performance, but not patients with PD and visual hallucination, had a significant reduction in fiber density in the left IFOF. The different results concerning the IFOF in PD patients between their study and ours might be the result of different research methods and patient groups.

In this study, there was a significant lateralization in the disruption of ILF integrity in the PD‐VH group, whereby only the left side was involved. While the mechanism of VH is not yet clear, previous studies have shown a left hemispheric involvement in PD‐HV. For example, neuroimaging studies have shown hypometabolism and decreased cerebral blood flow in the left hemisphere in patients with PD‐HV (Boecker et al., 2007; Okada et al., 1999). A structural MRI study reported that the brain of patients with PD‐VH showed a significant left hemispheric cortical atrophy in the prefrontal cortex, inferior occipital gyrus, and fusiform gyrus (Watanabe et al., 2013). Volumetric and DTI studies have reported there to be a lower FA value in the left optic nerve and atrophy of the lateral geniculate body, especially in the left side of the brain, in patients with PD‐VH compared with PD‐non‐VH patients (Lee et al., 2016). Moreover, one DTI study reported that disruption of white matter integrity in the left ILF was associated with VH in patients with schizophrenia (Ashtari et al., 2007). Therefore, hemispheric lateralization seems to be important in the genesis of VH in PD.

Several limitations of this study should be acknowledged. First, the number of patients in the PD‐VH group was smaller than those in the PD‐non‐VH group. Given that 20%‐75% patients with PD have VH (Diederich et al., 2009) and this study enrolled consecutive patients with PD, the proportion of patients with PD‐VH was smaller than PD‐non‐VH patients. Second, cognitive function was assessed using only the MMSE. It has been reported that VH in PD might be associated with frontal dysfunction (Onofrj et al., 2013; Ramírez‐Ruiz et al., 2008), which cannot be assessed using the MMSE. Therefore, to evaluate the association between VH in PD and cognitive dysfunction, it will also be necessary to examine frontal function using the Frontal Assessment Battery, for example.

In conclusion, our results indicate that disruption in the left ILF could play an important role in the genesis of VH in PD, independent of cognitive impairment and disease duration. While this study contributes to our understanding of VH in PD, the mechanisms underlying disruption in the ILF are unclear. Further studies should be conducted to clarify the association between disruption in the ILF and neuropathological increases in Lewy body deposits in the temporal lobes, especially in amygdala.

## CONFLICT OF INTEREST

No authors have any conflicts of interest to declare.

## AUTHOR CONTRIBUTIONS

Drs. Yoshioka and Yuki designed the study, drafted the manuscript, processed the statistical analysis, and reviewed the results. All authors collected and evaluated clinical data, processed the image analysis, reviewed the study design, and edited the manuscript.

### Peer Review

The peer review history for this article is available at https://publons.com/publon/10.1002/brb3.1883.

## Data Availability

The data that support the findings of this study are available from the corresponding author upon reasonable request.
